# Allelic Interference in Prion Replication Is Modulated by the Convertibility of the Interfering PrP^C^ and Other Host-Specific Factors

**DOI:** 10.1128/mBio.03508-20

**Published:** 2021-03-16

**Authors:** Juan Carlos Espinosa, Olivier Andreoletti, Alba Marín-Moreno, Severine Lugan, Patricia Aguilar-Calvo, Hervé Cassard, Patricia Lorenzo, Jean-Yves Douet, Ana Villa-Díaz, Naima Aron, Irene Prieto, Alvina Huor, Juan María Torres

**Affiliations:** a Centro de Investigación en Sanidad Animal (CISA-INIA), Madrid, Spain; b INRA, UMR 1225, Interactions Hôtes Agents Pathogènes, Ecole Nationale Vétérinaire de Toulouse, Toulouse, France; National Institutes of Health

**Keywords:** BSE, prion interference, prion propagation, prion replication, prion strain, scrapie

## Abstract

Early studies in transgenic mouse lines have shown that the coexpression of endogenous murine prion protein (PrP^C^) and transgenic PrP^C^ from another species either inhibits or allows the propagation of prions, depending on the infecting prion strain and interacting protein species. The way whereby this phenomenon, so-called “interference,” is modulated remains to be determined. In this study, different transgenic mouse lines were crossbred to produce mice coexpressing bovine and porcine PrP^C^, bovine and murine PrP^C^, or murine and porcine PrP^C^. These animals and their respective hemizygous controls were inoculated with several prion strains from different sources (cattle, mice, and pigs) to examine the effects of the simultaneous presence of PrP^C^ from two different species. Our results indicate interference with the infection process, manifested as extended survival times and reduced attack rates. The interference with the infectious process was reduced or absent when the potentiality interfering PrP^C^ species was efficiently converted by the inoculated agent. However, the propagation of the endogenous murine PrP^Sc^ was favored, allowing us to speculate that host-specific factors may disturb the interference caused by the coexpression of an exogenous second PrP^C^.

## Abstract (precis)

Prion propagation can be interfered with by the expression of a second prion protein in the host. In the present study, we investigated prion propagation in a host expressing two different prion protein genes.

## INTRODUCTION

Transmissible spongiform encephalopathies (TSEs) are fatal neurodegenerative diseases that affect humans and animals. TSEs are also called prion diseases because the causal agents are infectious particles essentially composed of a misfolded isoform (PrP^Sc^) of the cellular prion protein (PrP^C^) (1, 2). PrP^Sc^ is propagated via a template-assisted process involving physical interaction between the PrP^Sc^ template and the PrP^C^ substrate rendering a structurally modified PrP^Sc^ with a higher β-sheet content, which is prone to aggregation (3). PrP^Sc^ was originally defined according to its relative protease resistance and detergent insolubility compared with normal PrP^C^ (4, 5). However, disease-related forms of PrP^Sc^ that are protease sensitive have been described (6, 7). Distinct prion strains have been described. These strains are not encoded by differences in the PrP primary structure but show distinct disease phenotypes when transmitted to the same host, such as PrP^Sc^ biochemical features, distributions of prion deposits, clinical symptoms, and survival times (8).

PrP^C^ conversion into PrP^Sc^ is a posttranslational process. The molecular mechanisms underlying transmission of the strain-specific features of PrP^Sc^ are still unclear. It has been demonstrated that although originating from the same host, the PrP^Sc^ molecules of different prion strains vary in conformation and/or composition (9). Understanding PrP^C^-PrP^Sc^ interaction is a key step to elucidate the molecular mechanisms of prion propagation. Differences in the primary PrP^Sc^ amino acid sequence may alter the ability of a specific PrP^C^ to be efficiently converted into PrP^Sc^. Hence, a heterologous PrP^C^ may be conversion incompetent and thus could interfere with the conversion of a coexisting homologous—conversion-competent—PrP^C^. It has been proposed that interactions between dissimilar PrP^C^ and PrP^Sc^ molecules could slow down the aggregation and deposition of PrP^Sc^ by impairing interactions between homologous PrP monomers (10). This phenomenon is known as transdominant inhibition (11). Moreover, in a TSE-affected brain, different prion conformers may coexist and undergo competitive selection during replication, where the faster replication subset of conformers may be progressively selected (12).

Bovine spongiform encephalopathy (BSE), a TSE that affects cattle, was first reported in 1980 in the United Kingdom but soon attained epidemic proportions in several other European countries (13). The experimental finding that variant Creutzfeldt-Jacob disease (vCJD) diagnosed in humans was caused by BSE prions led to a major human and animal health crisis (14–16). The BSE agent has demonstrated a particularly good capacity to cross species barriers. Thus, besides humans, BSE has been transmitted to a range of zoo animals, cats (17–19), and goats (20, 21), while preserving its strain-specific signature (22, 23). Moreover, two more BSE strains have been described. These strains, called L-type BSE (24) and H-type BSE (25) due to their respective low and high electrophoretic mobilities compared to epidemic BSE, are also known as atypical BSE agents. Similarly, several scrapie strains have been identified (26–29). Different prion strains present different levels of transmissibility to another species. Therefore, in prion transmission between different species, both the strain and the PrP sequence of the recipient host are primary determinants of the species barrier (also called strain barrier) (30). However, while PrP is the major determinant for prion propagation, additional species-specific factors may have an influence on the prion propagated in a host-dependent manner (31, 32).

Transgenic mice expressing the PrP^C^ of different species are good experimental models of prion transmission (33, 34). Early experiments in one transgenic mouse line expressing both endogenous murine PrP^C^ and genetically engineered hamster PrP^C^ (35) revealed that the inoculation of these mice with hamster-adapted scrapie produced a prion infection characteristic of hamsters. This was the first evidence of the pivotal role of the PrP^C^ species in the prion infectious event. Nevertheless, the expression of endogenous murine PrP^C^ in the hamster transgenic mice allowed the propagation of mouse or hamster prions, suggesting the compatibility of both mouse and hamster PrP sequences in the replication of the infectious agent. Although the transgenic mice were able to produce both hamster and mouse prions, they were found to selectively produce one or the other, depending on the inoculum used (36). Prion infection studies are generally performed using transgenic mice that express the PrP^C^ of a particular species in a context of murine PrP knockout (KO). In fact, early studies in transgenic mice overexpressing human PrP^C^ showed that these mice were only efficiently infected with the sporadic form of Creutzfeldt-Jacob disease in the absence of murine PrP^C^ expression (37).

The present study was designed to further explore the effects of the simultaneous presence of PrP^C^ from two different species on prion replication. To this aim, a collection of transgenic mice expressing different combinations of bovine, porcine, and murine PrP^C^ were inoculated with prions from different sources. In these combinations, the inoculated PrP^Sc^ was either identical to one of the expressed PrP^C^ proteins in the mouse or not. Furthermore, the influence of host-specific factors on prion propagation in the presence of PrP^C^ of two different species was evaluated, as only murine PrP^C^ is in its natural host (the mouse), while either bovine or porcine PrP^C^ is not.

## RESULTS

To examine the effect of the simultaneous presence of PrP^C^ from two different species on prion replication, we produced transgenic mice expressing different pairs of PrP^C^: (i) murine and bovine PrP^C^ (TgMo/TgBo mice), (ii) murine and porcine PrP^C^ (TgMo/TgPo mice), and (iii) bovine and porcine PrP^C^ (TgPo/TgBo mice). Transgenic mouse lines PoPrP-Tg001, BoPrP-Tg110 and Tga20 were used to generate the animals coexpressing two different PrP^C^ proteins (Table 1). The brain PrP^C^ expression levels are similar for the BoPrP-Tg110 and Tga20 mouse lines but lower in the case of PoPrP-Tg001 mouse line (see Fig. S1 in the supplemental material). The newly generated heterozygous mice and their hemizygous controls (TgMo/−, TgBo/−, and TgPo/−) were selectively inoculated with cattle, mouse, sheep, or pig BSE, and disease transmissibility in each PrP^C^ context was determined. In addition to BSE, L-type BSE and sheep scrapie inocula were used to examine whether the results obtained with the BSE strain could be extended to other TSE agents. Brains of uninoculated C57BL/6 mice were used as negative inoculation controls in both the first and second passages. Neither shortening of the survival time of the different mouse lines used in this work (over 700 days) nor brain protease-resistant PrP (PrP^res^) positivity was observed after inoculation with the mentioned negative controls.

**TABLE 1:** Description of the mice used in the study

Genotype	PrP^C^ expressed	Expression level^*a*^	Abbreviation used in text	Description
mu*Prnp^+/+^*	Murine	10×	TgMo	Tga20 mouse line (47)
mu*Prnp^+/+^*	Murine	1×	C57BL/6	Conventional mouse
bo*Prnp^+/+^*	Bovine	8×	TgBo	BoPrP-Tg110 mouse line (46)
po*Prnp^+/+^*	Porcine	4×	TgPo	PoPrP-Tg001 mouse line (38)
KO*Prnp^−/−^*	Murine	0	*Prnp^−/−^*	PrP knockout (*Prnp*^−/−^) (50)
mu*Prnp^+/−^*	Murine	5×	TgMo/−	Progeny Tga20 (mu*Prnp^+/+^*) × PrP knockout (*Prnp*^−/−^)
bo*Prnp^+/−^*	Bovine	4×	TgBo/−	Progeny BoPrP-Tg110 (bo*Prnp^+/+^*) × PrP knockout (*Prnp*^−/−^)
po*Prnp^+/−^*	Porcine	2×	TgPo/−	Progeny PoPrP-Tg001 (po*Prnp^+/+^*) × PrP knockout (*Prnp*^−/−^)
mu*Prnp^+/−^*	Murine	0.5×	C57BL/6/−	Progeny C57BL/6 × PrP knockout (*Prnp*^−/−^)
mu*Prnp^+/−^*, bo*Prnp^+/−^*	Murine and bovine		C57BL/6/TgBo	Progeny C57BL/6 × BoPrP-Tg110
mu*Prnp^+/−^*, bo*Prnp^+/−^*	Murine and bovine		TgMo/TgBo	Progeny TgMo × BoPrP-Tg110
po*Prnp^+/−^*, bo*Prnp^+/−^*	Porcine and bovine		TgPo/TgBo	Progeny PoPrP-Tg001 × BoPrP-Tg110
mu*Prnp^+/−^*, po*Prnp^+/−^*	Murine and porcine		TgMo/TgPo	Progeny TgMo × PoPrP-Tg001

a Relative to the PrP^C^ expression level in the indicated species.

10.1128/mBio.03508-20.1 FIG S1 Comparative brain PrP^C^ expression in TgBo, TgMo, and TgPo mice. (A) Immunoblot of brain PrP^C^ expression detected with the 12B2 MAb. The 12B2 MAb is specific for epitope _93_WGQGG_97_ (numbered according to porcine sequence) present in the murine, bovine, and porcine PrP. Whole brains were homogenized as 10% brain homogenates in extraction buffer (0.5% NP-40, 1% sodium deoxycholate, 10 mM EDTA in phosphate-buffered saline at pH 7.4, and the Complete cocktail of protease inhibitors from Roche). Samples were precleared by centrifugation (2,000 × *g* for 5 min), after which an equal volume of 2× SDS reducing sample loading buffer was added to the samples, and the mixture was boiled for 5 min before loading on 12% Bis-Tris gel. Ten microliters of undiluted samples and 1/2 dilutions (in an equal volume of 1× SDS reducing sample loading buffer) were loaded onto 12% Bis-Tris gels. The figure illustrates a representative set of three independent experiments. (B) Brain PrP^C^ expression was quantified, and the diagrams illustrate the mean densitometric values from three independent experiments. Data from TgPo brains were always standardized as 1 relative unit. Error bars represents the standard deviation of the mean value. MW, molecular weight in kilodaltons. Download FIG S1, TIF file, 0.5 MB. Copyright © 2021 Espinosa et al. 2021 Espinosa et al. https://creativecommons.org/licenses/by/4.0/ This content is distributed under the terms of the Creative Commons Attribution 4.0 International license.

Two different questions have been addressed: (i) how the presence of a heterologous PrP^C^ species affects prion propagation of the homologous PrP^C^ with the same amino acid sequence than the inoculated PrP^Sc^ and (ii) how the coexpression of two different PrP^C^ species affects the propagation of a PrP^Sc^ from a third species. To quantify the effect of the interference, we have incorporated a single parameter, the interference score (IS), as a measure of the interference with PrP^Sc^ propagation by the presence of an interfering PrP (see Materials and Methods).

### Prion propagation in the presence of a heterologous PrP^C^ species. (i) BSE agent propagation in a murine and bovine PrP^C^-coexpressing host.

In this case, animals expressing murine PrP^C^ in addition to bovine PrP^C^ were generated and compared with those expressing either murine nor bovine PrP^C^. It should be highlighted that in animals coexpressing murine and bovine PrP^C^, while murine PrP^C^ is in the context of its natural host (the mouse), bovine PrP^C^ is not.

**FIG 1:**
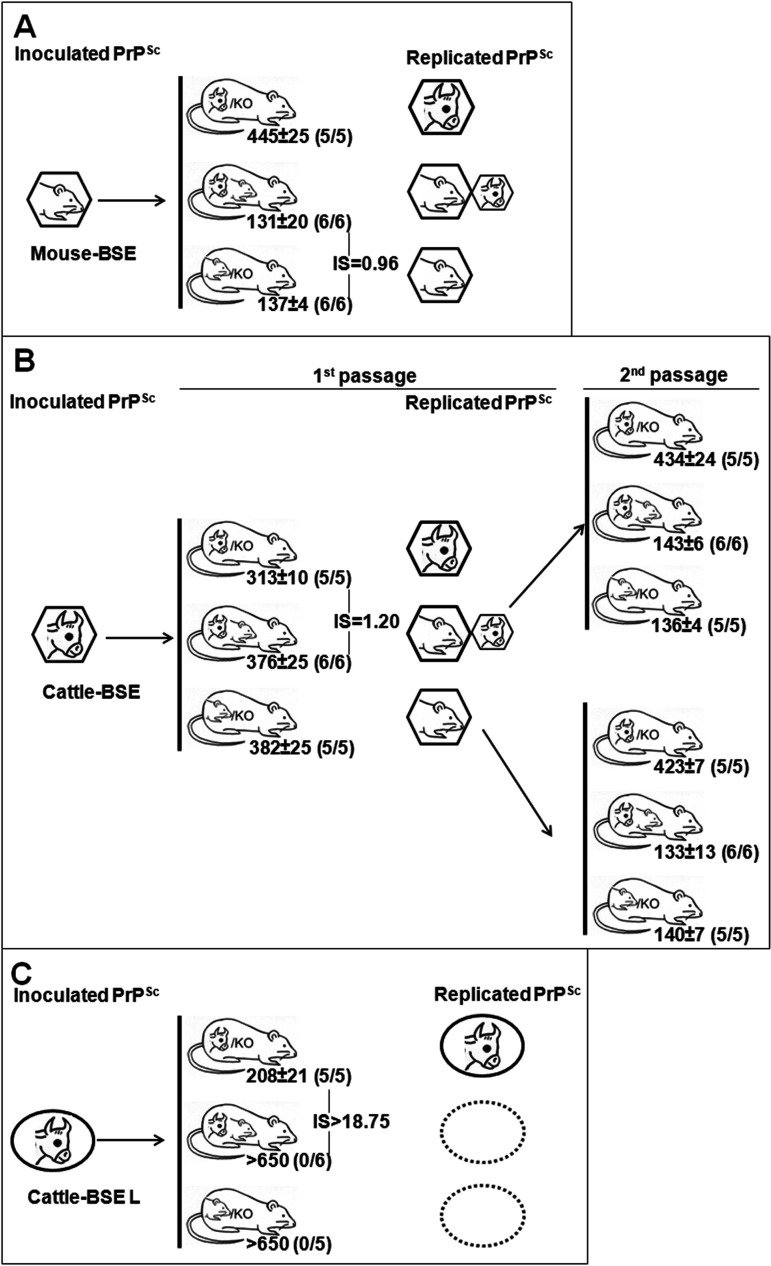
Transmission of mouse BSE (A), cattle BSE (B), or cattle BSE-L (C) after intracerebral inoculation in TgMo/−, TgBo/−, and TgMo/TgBo mice. The mean survival time in days postinoculation ± standard deviation (SD) is shown. *n*/*n*_0_, number of diseased PrP^res^-positive animals/inoculated animals. IS, interference score of the interfering PrP. PrP^Sc^ species are depicted as hexagons for classical BSE or ellipses for atypical BSE-L.

When mouse BSE was used as the inoculum, the survival times of the inoculated mice expressing murine PrP^C^ (TgMo/−) or both murine and bovine PrP^C^ (TgMo/TgBo) were not significantly different (*P* = 0.5222 [Fig. 1A]), indicating no effects on the replication of mouse BSE when bovine PrP^C^ is coexpressed. When cattle BSE was inoculated (Fig. 1B), heterozygous TgMo/TgBo mice showed no significant differences in survival times compared to animals expressing only murine PrP^C^ (*P* = 0.7127). However, a slightly longer survival time (*P* = 0.0002) was observed in these TgMo/TgBo mice compared to those observed in mice expressing only bovine PrP^C^ (TgBo/−). This difference seems to be lower than expected—probably due to the small variation in survival times between hemizygous TgBo/− and TgMo/− mice when inoculated with cattle BSE. This slight difference (IS = 1.2) suggests that the mouse allele only weakly interferes with conversion of the bovine allele. However, an alternative interpretation is also possible as TgMo/TgBo mice could succumb from conversion of mouse PrP, thus, reflecting the efficiency of conversion of murine PrP but not of bovine PrP.

Strikingly, whatever the PrP^Sc^ present in the inoculum (mouse or cattle), survival times in heterozygous TgMo/TgBo mice were similar to those observed in hemizygous TgMo/− mice.

**FIG 2:**
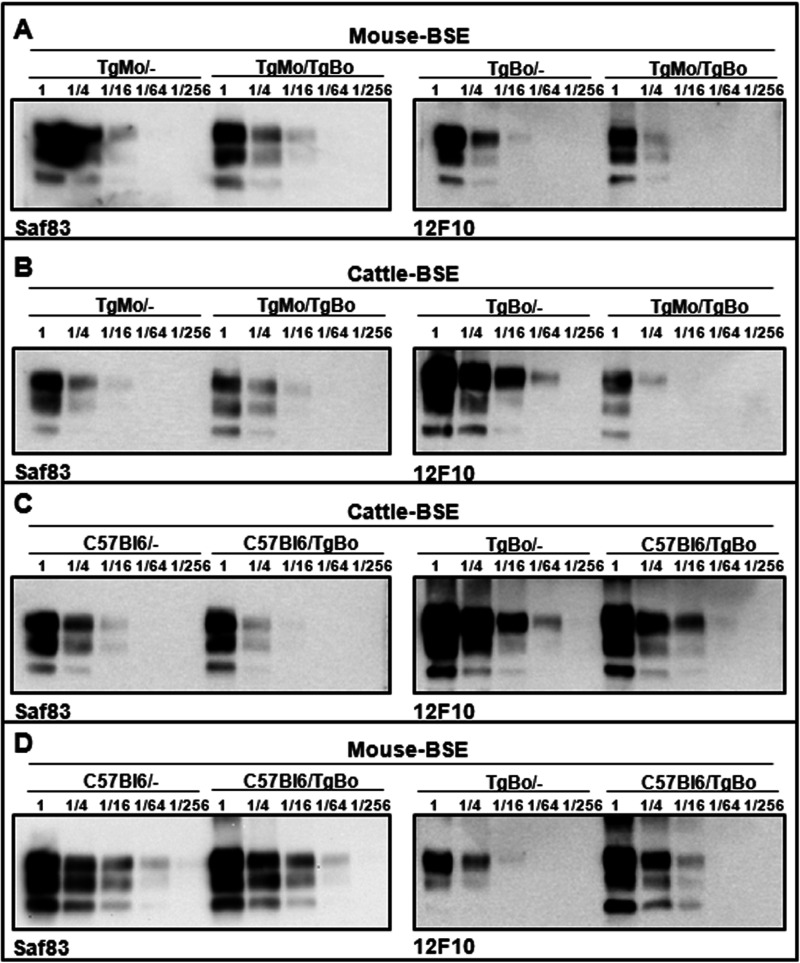
Brain PrP^res^ in inoculated mice. Shown are immunoblots of PrP^res^ from brain detected with either the Saf83 (left panels) or 12F10 (right panels) MAb. Direct samples (2-mg equivalent of 10% brain homogenates) and 1/4 dilutions were loaded onto 12% Bis-Tris gels. The results shown are representative of at least two independent experiments. (A to D) Brain PrP^res^ from mice inoculated with mouse BSE in TgMo/−, TgMo/TgBo, and TgBo/− mice (A), cattle BSE in TgMo/−, TgMo/TgBo, and TgBo/− mice (B), cattle BSE in C57BL/6/−, C57BL/6/TgBo, and TgBo/− mice (C), and mouse BSE in C57BL/6/−, C57BL6/TgBo, and TgBo/− mice (D).

In order to assess the impact of coexpression of murine and bovine PrP^C^ on the PrP^Sc^ propagation process, immunoblotting using two antibodies that specifically probe the murine (SAF83) or the bovine (12F10) PrP were used to estimate the levels of brain PrP^res^ accumulation in the inoculated mice. Whatever the origin of the BSE inoculum (mouse or cattle), the SAF83 PrP^res^ signals observed in clinically affected TgMo/− and TgMo/TgBo mice were similar (Fig. 2A and B). Conversely, in cattle BSE-inoculated TgMo/TgBo mice, the 12F10 PrP^res^ signal was at least 16 times weaker than that in clinically affected TgBo/− animals (Fig. 2B). Since in cattle BSE-inoculated TgMo/TgBo mice the survival time was only 1.2-fold longer than that in TgBo/− mice, the survival time cannot explain the lower bovine PrP^res^ accumulation level observed in TgMo/TgBo mouse brain. In addition, cattle BSE passaged in TgMo/− or TgMo/TgBo mice was used to inoculate groups of TgMo/−, TgBo/−, and TgMo/TgBo mice (Fig. 1B). In both cases, the incubation periods recorded in the three mouse groups showed a similar pattern: a short survival time in both TgMo/− and TgMo/TgBo mice and a prolonged survival time in TgBo/− mice. These results clearly differed from those observed in mice inoculated with cattle BSE, suggesting that, in TgMo/TgBo mice inoculated with cattle BSE, the murine allele is predominantly being propagated. In all cases, BSE-inoculated TgMo/TgBo mice showed the same survival time as the TgMo/− control, and therefore, mouse BSE is actively replicating the mouse allele without apparent interference by the bovine allele, while the replication of cattle BSE in the bovine allele is interfered with by the presence of the murine allele, which is finally predominantly propagated, as observed by bioassay and brain PrP^res^ accumulation.

To assess the relevance of the inoculation route in the outcome of the experiment, TgMo/TgBo, TgMo/−, and TgBo/− mice were intraperitoneally inoculated with cattle or mouse BSE. In both cases, intraperitoneally inoculated TgMo/TgBo mice died with a similar survival time pattern (Table 2) compared with the pattern previously observed following inoculation by the intracerebral route (Fig. 1A and B). As previously observed by the intracerebral route, survival times in heterozygous TgMo/TgBo mice were similar to those observed in hemizygous TgMo/− mice whatever the PrP^Sc^ present in the inoculum (mouse or cattle).

**TABLE 2:** Intraperitoneal inoculation of BSE isolates in mice overexpressing murine and bovine PrP

Mice	Mean survival time, dpi (*n*/*n*_0_)^*a*^	
	Cattle BSE	Mouse BSE
TgMo/−	679 ± 57 (6/6)	279 ± 9 (6/6)
TgMo/TgBo	709 ± 70 (6/6)	272 ± 15 (6/6)
TgBo/−	457 ± 54 (6/6)	>650 (6/6)

a Survival time is indicated as mean number of days postinoculation (dpi) ± SD for all the mice that scored positive for PrP^res^. *n*/*n*_0_, number of diseased PrP^res^-positive animals/inoculated animals.

In TgMo/TgBo mice, a higher expression of the murine versus bovine PrP (Table 1) could be the cause of the effect observed in TgMo/TgBo mice. However, no significant differences were observed when PrP^C^ expression levels from TgBo/− and TgMo/− brains were compared (Fig. S1). To test the influence of PrP^C^ expression levels, we inoculated heterozygous C57BL/6/TgBo mice with mouse BSE and cattle BSE. In these mice, the expression of bovine PrP^C^ is significantly higher than that of mouse PrP. After inoculation with mouse BSE, 100% of the mice were infected, but the survival time in TgBo/− mice was longer than those in C57BL/6/TgBo or C57BL/6/− mice (*P* < 0.0001 [Table 3]). This result suggests a slight effect of the expression of bovine PrP^C^ on the homologous replication of the murine allele. On the other hand, survival times in cattle BSE-inoculated C57BL/6/TgBo mice were considerably longer than those in TgBo/− mice (Table 3). In contrast, C57BL/6/− mice inoculated with cattle BSE died at the end of their life span without showing clinical signs of neurological disease, but when their brains were analyzed, 100% of them were found PrP^res^ positive (Table 3).

**TABLE 3:** Intracerebral inoculation of cattle and mouse BSE isolates in C57BL/6/TgBo mice

Mice	Mean survival time, dpi (*n*/*n*_0_)^*a*^	
	Cattle BSE	Mouse BSE
C57BL/6/−	>650 (6/6)	304 ± 8 (6/6)
C57BL/6/TgBo	455 ± 11 (6/6)	333 ± 7 (6/6)
TgBo/−	313 ± 10 (5/5)	445 ± 25 (5/5)

a Survival time is indicated as mean number of days postinoculation (dpi) ± SD of all the mice scored positive for PrP^res^. *n*/*n*_0_, number of diseased PrP^res^-positive animals/inoculated animals.

In C57BL/6/TgBo mice inoculated with cattle BSE, the SAF83 immunoblot indicated that the accumulation of murine PrP^res^ in the brain was similar to that in C57BL/6/− mice (Fig. 2C). The 12F10 immunoblot was consistent with the accumulation of quite similar amounts of bovine PrP^res^ in clinically affected C57BL/6/TgBo and TgBo/− mice. Mouse BSE-inoculated C57BL/6/TgBo mice displayed similar accumulation of murine and bovine PrP^res^ in their brain (as assessed by SAF83 and 12F10 immunoblots, respectively) compared to C57BL/6/− and TgBo/− mice, respectively (Fig. 2D). These data indicate that in animals that express significantly more bovine PrP^C^ (about 3×) than murine PrP^C^, the capability of murine PrP^C^ to interfere with the bovine PrP^Sc^ replication is slightly reduced compared to animals that express similar amounts of both bovine and murine PrP^C^ (Fig. 1B and 2B). These results suggest that differences in the bovine and murine PrP^C^ expression ratio can affect the observed interference.

### (ii) Atypical BSE-L agent propagation in a murine and bovine PrP^C^-coexpressing host.

To assess whether the observed interference effect is strain-specific, atypical BSE-L (cattle BSE-L) was used as the inoculum in TgMo/−, TgMo/TgBo, and TgBo/− mice (Fig. 1C). The cattle BSE-L agent was only able to replicate in TgBo/− mice, while both heterozygous TgMo/TgBo and hemizygous TgMo/− mice were resistant to the infection with this agent, and hence, the interference score was high (IS > 18). None of the animals succumbed to the disease, and when euthanized at the end of their life span, they showed no clinical signs or PrP^res^ in their brains. These results indicate that, contrary to epidemic BSE, the expression of the heterologous murine PrP^C^ prevents the replication of the bovine PrP^Sc^ in animals inoculated with cattle BSE-L, thus suggesting that the interference effect of a heterologous PrP^C^ on prion propagation is strain dependent and probably related to the inconvertibility of the interfering PrP^C^.

### (iii) BSE agent propagation in a murine and porcine PrP-coexpressing host.

**FIG 3:**
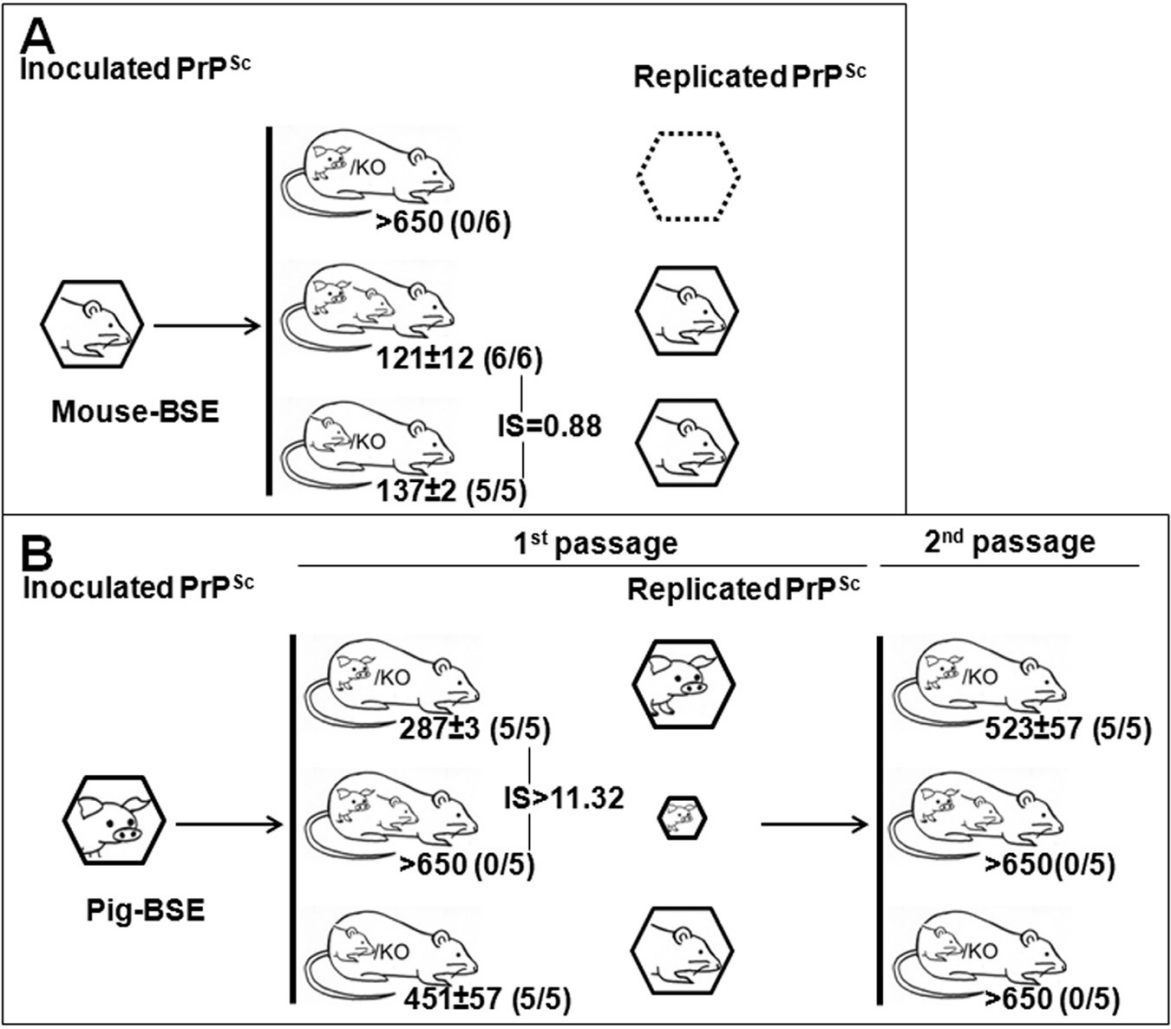
Transmission of mouse BSE (A) or pig BSE (B) after intracerebral inoculation in TgMo/−, TgPo/−, and TgMo/TgPo mice. The mean survival time in days postinoculation ± standard deviation is shown. *n*/*n*_0_, number of diseased PrP^res^-positive animals/inoculated animals. IS, interference score of the interfering PrP. PrP^Sc^ species are depicted as hexagons.

We also investigated the interference phenomenon in animals coexpressing the murine PrP^C^ (in the context of its natural host) beside the porcine PrP^C^ sequence (Fig. 3). Inoculation of mouse BSE in TgPo/− mice was inefficient, as none of the mice was scored positive for the transmission of the disease, while it readily infected TgMo and TgMo/TgPo mice (Fig. 3A). Statistical analysis confirms that there is no interference (*P* < 0.0001). Even the onset of the disease is very slightly accelerated in the animals expressing both alleles, showing an interference score of around 1, indicating that the presence of the porcine allele, despite its inconvertibility, does not affect the replication of the murine allele.

When pig BSE was used as the inoculum (Fig. 3B), 100% of hemizygous TgPo/− and TgMo/− mice were infected, while none of the heterozygous TgMo/TgPo mice was scored positive for the disease, suggesting a dual interference effect, as supported by the elevated interference score observed for these transmissions (IS > 11.32). The second passage of brains from TgMo/TgPo mice inoculated with pig BSE revealed a lack of infectivity in TgMo mice, and only residual infectivity could be detected in TgPo mice (Fig. 3B).

### (iv) BSE agent propagation in a bovine and porcine PrP-coexpressing host.

**FIG 4:**
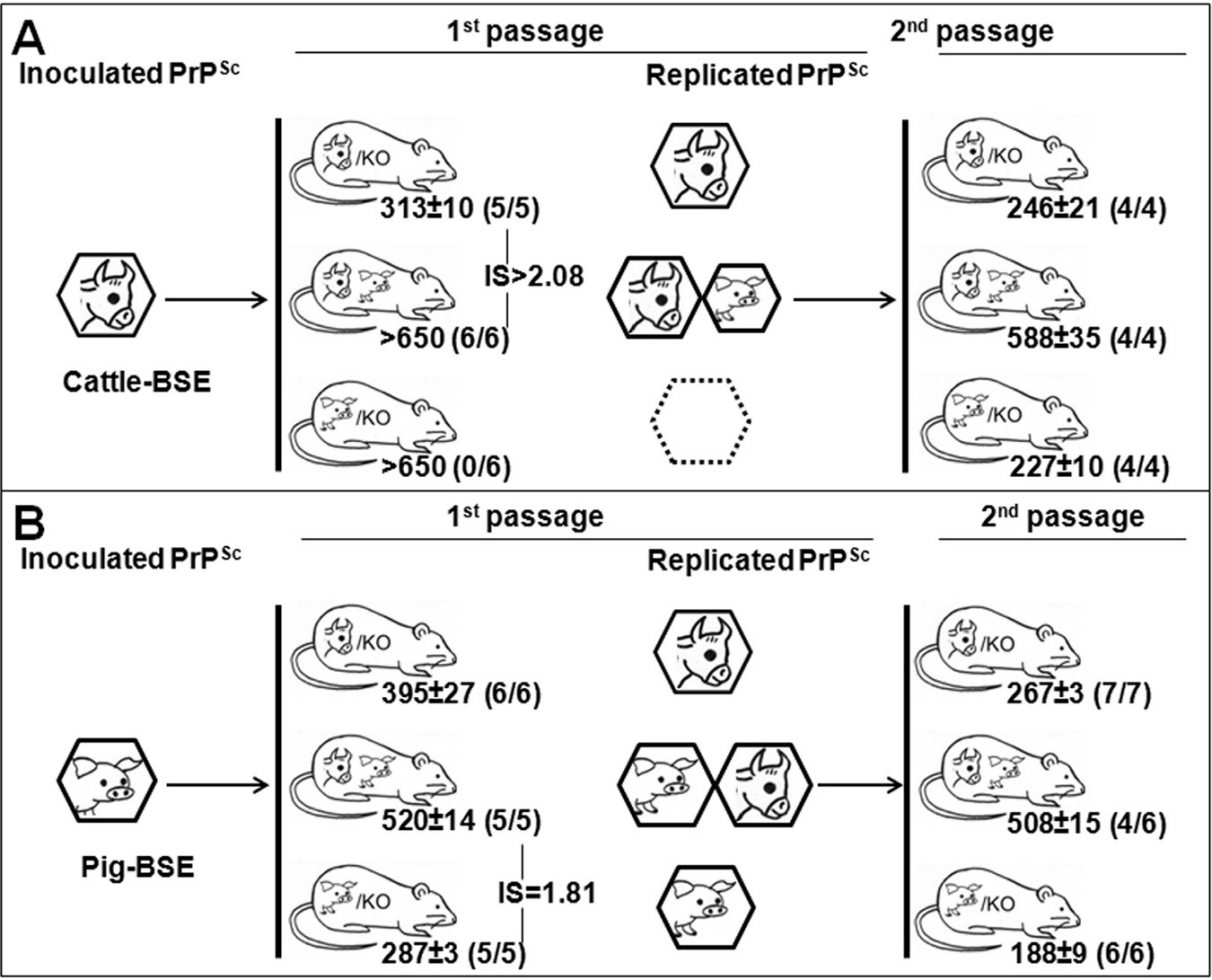
Transmission of cattle BSE (A) or pig BSE (B) after intracerebral inoculation in TgBo/−, TgPo/−, and TgBo/TgPo mice. The mean survival time in days ± SD is shown. *n*/*n*_0_, number of diseased PrP^res^-positive animals/inoculated animals. IS, interference score of the interfering PrP. PrP^Sc^ species are depicted as hexagons.

**FIG 5:**
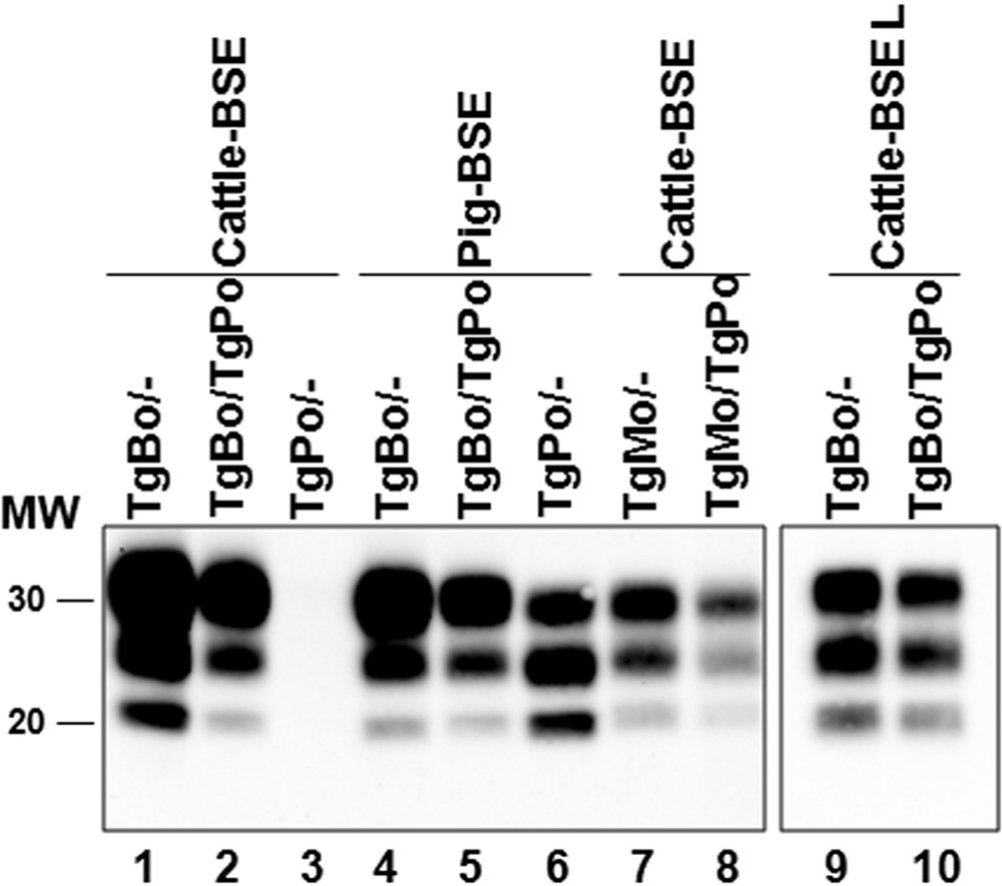
Brain PrP^res^ in inoculated mice. Shown is an immunoblot of brain PrP^res^ detected with the Sha31 MAb. Shown are brain PrP^res^ proteins from TgBo/−, TgBo/TgPo, and TgPo/− mice inoculated with cattle BSE (lanes 1, 2, and 3, respectively), TgBo/−, TgBo/TgPo, and TgPo/− mice inoculated with pig BSE (lanes 4, 5, and 6, respectively), TgMo/− and TgMo/TgPo mice inoculated with cattle BSE (lanes 7 and 8, respectively), and TgBo/− and TgBo/TgPo mice inoculated with cattle BSE-L (lanes 9 and 10, respectively). Lane 3 was included as a negative control. From a 0.5- to 2-mg equivalent of 10% brain homogenate was loaded per lane in order to obtain similar quantities of PrP^res^ in each lane for better comparison. MW, molecular weight in kilodaltons.

Further analyses were accomplished in transgenic mice coexpressing bovine and porcine PrP (Fig. 4). It should be noted that in this case, none of the expressed PrP^C^ is in the context of its natural host. Cattle BSE was not transmitted in hemizygous TgPo/− mice (Fig. 4A). Interestingly, while transgenic mice expressing only bovine PrP^C^ (TgBo/−) were readily infected with cattle BSE, none of the animals coexpressing bovine and porcine PrP^C^ showed evident clinical signs, yet they scored positive for the presence of PrP^res^ in their brains when culled after 650 days postinfection (dpi). The PrP^res^ profile obtained from TgBo/TgPo brain extracts was indistinguishable from those obtained from TgBo/− brains (Fig. 5). Hence, porcine PrP^C^ seems to entail a strong interfering effect on bovine PrP^Sc^ propagation in TgBo/TgPo mice inoculated with cattle BSE. To investigate whether PrP^Sc^ propagation is restricted to bovine PrP, brain homogenates from cattle BSE-inoculated TgBo/TgPo transgenic mice were reinoculated into TgPo, TgBo, and TgBo/TgPo mice. As shown in Fig. 4A, the totalities of both TgBo- and TgPo-inoculated mice were scored positive for the disease, with short survival times, as previously described for the infection with cattle BSE in TgBo mice and pig BSE in TgPo mice (23). Remarkably, TgBo/TgPo mice were 100% susceptible to this cattle BSE passaged in the TgBo/TgPo transgenic mouse inoculum but showed significantly (*P* < 0.0001) longer survival times (588 ± 35 dpi) than those observed in TgBo/− mice inoculated with cattle BSE (313 ± 10 dpi) or TgPo/− mice inoculated with pig BSE (287 ± 3 dpi). These second passages suggest that both bovine and porcine PrP^Sc^ were generated in the brains of TgBo/TgPo mice inoculated with cattle BSE (1st passage), while their simultaneous replication in the second passage of TgBo/TgPo mice was impaired by the presence of the other prion protein.

When pig BSE was used as the inoculum, TgBo/− and TgPo/− mice were infected without evidence of a transmission barrier, as previously described (38). As shown in Fig. 4B, heterozygous TgBo/TgPo mice were also 100% susceptible to the inoculation of pig BSE, but again, the manifestation of the disease was delayed (*P* < 0.0001) compared to their hemizygous counterparts, the TgBo/− and TgPo/− mice. Similar behavior was maintained after the second passage of pig BSE-infected TgBo/TgPo brains in TgBo/TgPo mice. They showed survival times longer than 500 dpi, and only four out of six animals scored positive for PrP^res^ in their brains. Again, the PrP^res^ profile obtained in brain extracts from TgBo/TgPo animals was indistinguishable from that observed in TgBo/− brains (Fig. 5). In addition, TgBo and TgPo mice were 100% susceptible to brain homogenate from pig BSE passaged in TgBo/TgPo mice, supporting the coreplication of both bovine and porcine PrP^Sc^ during the first passage on TgBo/TgPo mice (Fig. 4B). These results suggest that bovine PrP^Sc^ and porcine PrP^Sc^ can replicate in TgBo/TgPo mice but less efficiently than separately (with an observed interference score of around 2 in both cases), indicating that the detrimental effect on PrP^Sc^ conversion mutually affects both PrP species.

### Prion propagation in a host expressing two PrP^C^ species different from the inoculated PrP^Sc^. (i) Cattle BSE in a murine and porcine PrP^C^-coexpressing host.

**FIG 6:**
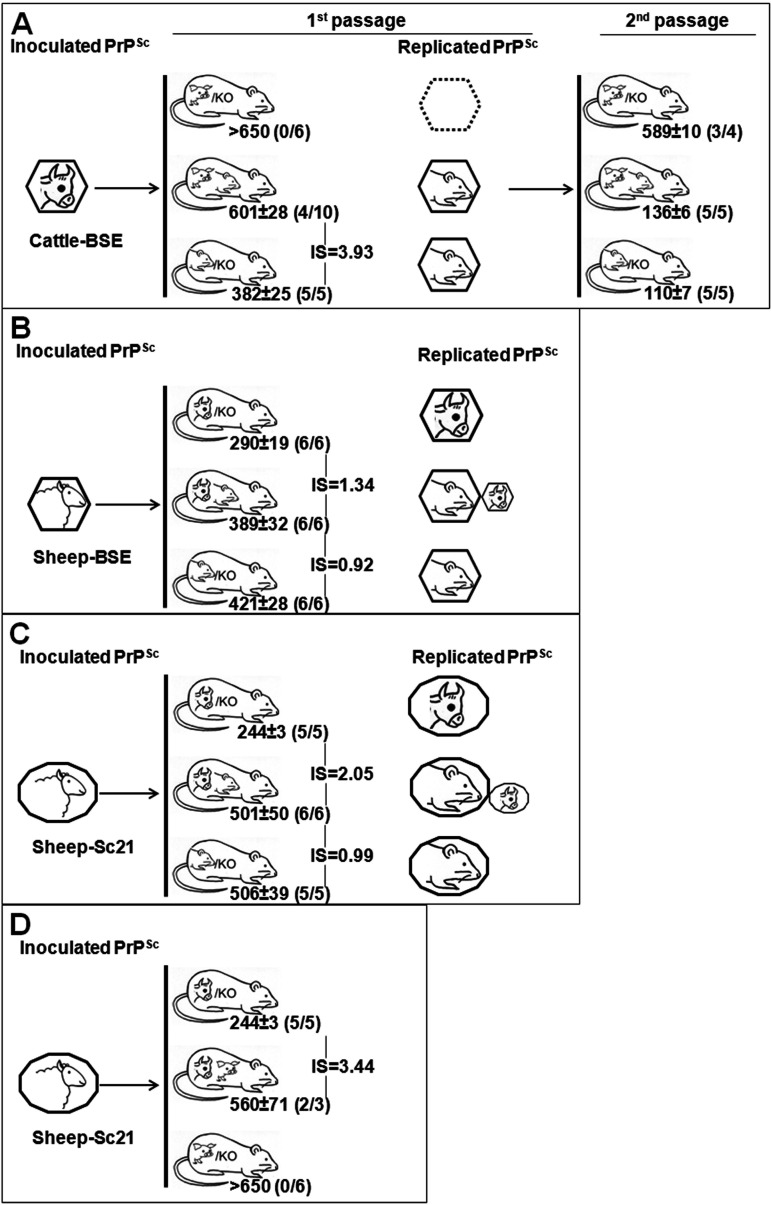
Transmissions in a host expressing PrP^C^ from two species different from the inoculated PrP^Sc^. Shown is intracerebral inoculation of cattle BSE in TgMo/−, TgPo/−, and TgMo/TgPo mice (A), sheep BSE in TgMo/−, TgBo/−, and TgMo/TgBo mice (B), sheep Sc21 in TgMo/−, TgBo/−, and TgMo/TgBo mice (C), and sheep Sc21 in TgBo/−, TgPo/−, and TgBo/TgPo mice (D). Shown is the mean survival time in days postinfection ± SD. *n*/*n*_0_, number of diseased PrP^res^-positive animals/inoculated animals. IS, interference score of the interfering PrP. PrP^Sc^ species are depicted as polygons; a dashed polygon indicates that PrP^Sc^ was not detected.

**FIG 7:**
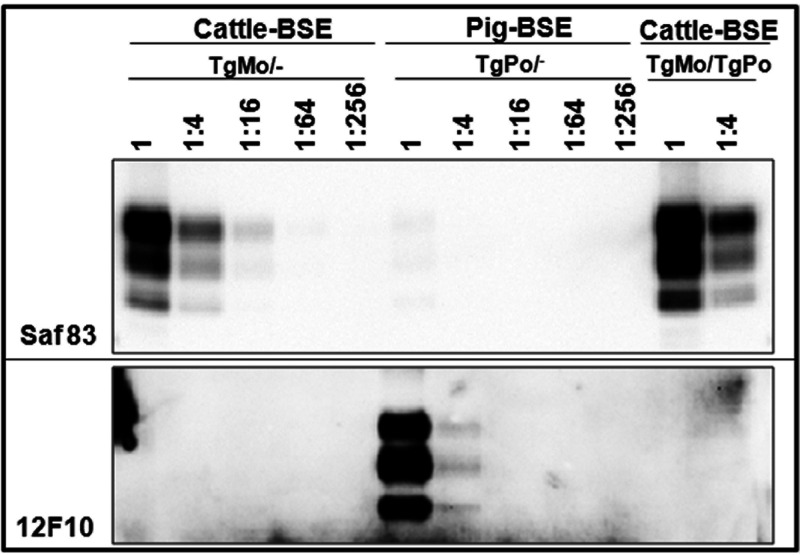
Brain PrP^res^ in inoculated mice. Shown is an immunoblot of brain PrP^res^ detected with either the Saf83 (top) or 12F10 (bottom) MAb. Direct samples (2-mg equivalent of 10% brain homogenates) and 1/4 dilutions were loaded onto 12% Bis-Tris gels. The results shown are representative of at least two independent experiments. Show is brain PrP^res^ from mice inoculated with cattle BSE in TgMo/− mice and TgMo/TgPo or pig BSE in TgPo/− mice.

Cattle BSE was inoculated in heterozygous TgMo/TgPo mice and their respective hemizygous controls. As mentioned before, cattle BSE was not able to infect TgPo/− mice, yet could infect TgMo/− mice, with attack rates of 100% (Fig. 6A). When inoculated into TgMo/TgPo mice, cattle BSE led to 40% attack rates, long survival times of around 600 dpi (rendering an interference score of around 4), and a PrP^res^ profile identical to that found in TgMo/− brains (Fig. 5). Brains from TgMo/TgPo mice inoculated with cattle BSE and scoring PrP^res^ positive were passaged a second time in TgMo, TgMo/TgPo, and TgPo mice. Short survival times were observed in both TgMo and TgMo/TgPo mice (110 ± 7 and 136 ± 6 dpi, respectively), showing a small but significant difference (*P* = 0.0003). TgPo mice became infected with an evident transmission barrier (survival for 589 ± 10 dpi and three out of four animals scoring positive for PrP^res^). A similar result was previously described for the inoculation of mouse BSE prions in TgPo mice (survival time of 506 dpi and one out of six mice scoring positive for PrP^res^) (23). Additional analysis of the PrP^res^ from TgMo/TgPo brains infected with cattle BSE evidenced that—as expected by the bioassay outcome—murine PrP^res^ is present, as detected with the Saf83 monoclonal antibody (MAb), while porcine PrP^res^ was not detected with the 12F10 MAb (Fig. 7).

Taken together, these results suggest that only the mouse PrP^Sc^ was replicated in TgMo/TgPo mice inoculated with cattle BSE. Nevertheless, mouse PrP^Sc^ replication is severely interfered with by porcine PrP, despite the relatively lower expression level of the pig PrP^C^ in comparison with mouse PrP^C^.

### (ii) Sheep BSE in a murine and bovine PrP-coexpressing host.

**FIG 8:**
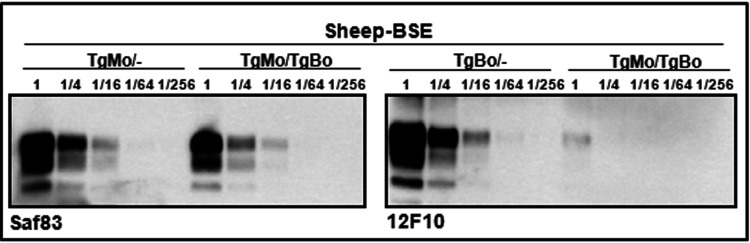
Brain PrP^res^ in inoculated mice. Shown is an immunoblot of PrP^res^ from brain detected with either the Saf83 (left) or 12F10 (right) MAb. Direct samples (2-mg equivalent of 10% brain homogenates) and 1/4 dilutions were loaded onto 12% Bis-Tris gels. The results shown are representative of at least two independent experiments. Show is brain PrP^res^ from mice inoculated with sheep BSE in TgMo/−, TgMo/TgBo, and TgBo/− mice.

BSE agent after adaptation in ARQ sheep (sheep BSE) was used as a heterologous inoculum in TgMo/TgBo mice. The observed outcome (Fig. 6B) was very similar to the results obtained after inoculation of cattle BSE into TgMo/TgBo mice (Fig. 1B), propagating efficiently in TgBo/− mice and with longer survival times in both TgMo/TgBo and TgMo/− mice. TgMo/TgBo and TgMo/− mice showed no significant differences in their survival times (*P* = 0.0963 [Fig. 6B]). As previously observed for the cattle BSE inoculum in these mice, although this slight difference (IS = 1.34) suggests that the mouse allele only weakly interferes with the conversion of the bovine allele, this result could also be interpreted as TgMo/TgBo mice having succumbed from the conversion of mouse PrP, thus, reflecting the efficiency of conversion of murine PrP but not of bovine PrP. The levels of SAF83 PrP^res^ signal observed in TgMo/− and TgMo/TgBo mice were similar, and the 12F10 PrP^res^ signal in the TgMo/TgBo mice was at least 16 times weaker than that in TgBo/− animals (Fig. 8).

### (iii) Sheep scrapie in a murine and bovine PrP-coexpressing host.

**FIG 9:**
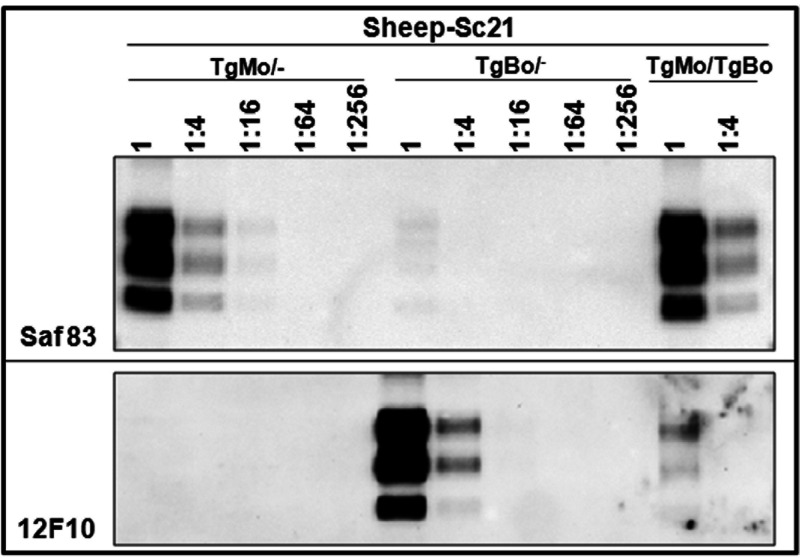
Brain PrP^res^ in inoculated mice. Shown is an immunoblot of brain PrP^res^ detected with either the Saf83 (top) or 12F10 (bottom) MAb. Undiluted samples (2-mg equivalent from 10% brain homogenates) and 1/4 dilutions were loaded onto 12% Bis-Tris gels. The results shown are representative of at least two independent experiments. Shown is brain PrP^res^ from mice inoculated with sheep Sc21 in TgMo/−, TgBo/−, or TgMo/TgBo mice.

In another set of experiments, a sheep scrapie isolate (Sc21) was used as the inoculum, with PrP^Sc^ different from the two PrP^C^ proteins coexpressed in the recipient. While TgMo/TgBo and TgBo/− mice inoculated with sheep Sc21 showed no significant differences in their long survival times (*P* = 0.6952 [Fig. 6C]), TgBo/− mice were readily infected with sheep Sc21, evidencing the interference with bovine PrP^Sc^ replication by the presence of mouse PrP^C^ (IS = 2.05). Biochemical analysis of PrP^res^ from TgMo/TgBo brains infected with sheep Sc21 confirms the interference with bovine PrP^Sc^ replication as mouse PrP^res^ was present in similar levels to TgMo/− mice, while only residual levels of bovine PrP^res^ could be detected (Fig. 9).

Sheep Sc21 was also transmitted to heterozygous TgBo/TgPo mice, although the attack rate was lower and the survival time longer than those in TgBo/− mice (IS = 3.44 [Fig. 6D]). Consistent with our prior observations (39, 40), scrapie was not transmitted to TgPo/− mice, and when euthanized at the end of their life span, they scored negative for PrP^res^. As mentioned before, the amino acid sequence of the inoculated PrP^Sc^ (ovine) is different from those of any of the PrP^C^ proteins expressed in the recipient (bovine and porcine). However, since the sheep Sc21 isolate readily infects TgBo/− mice, porcine PrP^C^ must be responsible for the interfering effect, increasing the survival times in TgBo/TgPo mice.

The results obtained when the species origin of the inoculated PrP^Sc^ is different from the two PrP^C^ proteins coexpressed in the transgenic mouse indicate that an interference effect can be observed but with a complex outcome.

## DISCUSSION

This study evaluates the potential interference with the PrP^Sc^ replication process by a PrP^C^ protein from a second species expressed in the recipient transgenic mouse model. The amino acid sequence differences between the donor PrP^Sc^ and the recipient PrP^C^ play an important modulatory role in the interspecies transmissibility of TSE agents (36). Furthermore, amino acid sequence differences in the second species PrP^C^ may be relevant in the interference with PrP^Sc^ replication (41). In this work, different combinations of PrP^C^ species pairs were challenged with PrP^Sc^ proteins from different sources (cattle, mice, and pigs) to examine the effects of the simultaneous presence of PrP^C^ from two different species.

In the first set of experiments, the amino acid sequence of the PrP^Sc^ inoculated was the same as that of the PrP^C^ expressed in the host (homologous PrP^C^). Thus, compared to the appropriate control, there is not any other factor affecting the transmissibility of the PrP^Sc^ inoculated than the second PrP^C^ expressed in the host (heterologous PrP^C^). As previously observed by using *in vitro* conversion (42), heterologous (less convertible or nonconvertible PrP^C^) may interact with PrP^Sc^, and as a consequence, the conversion of the homologous PrP^C^ may be interfered with. Moreover, the heterologous PrP^C^ expressed in the *in vivo* model may result in the new PrP^Sc^ counterpart, and accordingly, bidirectional interference may occur in the context of the mouse expressing PrP^C^ from two species. In the *in vivo* model used here (the mouse), murine PrP^C^ is expressed in its natural context, but bovine or porcine PrP^C^ is not. In this sense, the interaction of host-specific factors with the expressed PrP^C^ may affect the interference process.

Our transmission experiments using transgenic mice that coexpress an exogenous PrP^C^ show that the expression of bovine PrP^C^ at similar levels to murine PrP^C^ is not able to alter the disease caused by the inoculated TSE agent compared to animals expressing only murine PrP^C^ (Fig. 1). This was independent of (i) the route of inoculation used (intracranial or intraperitoneal) (Table 2), (ii) the PrP^Sc^ amino acid sequence inoculated (either from mice [Fig. 1A], cattle [Fig. 1B], or sheep [Fig. 6B and C]), and (iii) the tested prion strains from BSE (Fig. 1A and B and Fig. 6B) and sheep scrapie (Fig. 6C). Only the expression of higher levels of bovine PrP^C^ than murine PrP^C^ is able to alter the disease caused by the inoculated TSE agent when murine PrP^C^ is expressed alone (Table 3), evidencing that PrP expression levels are relevant in the interference process. Remarkably, in all these experiments, murine PrP^C^ is coexpressed in the context of its natural host. In contrast, the coexpression of either bovine or murine PrP^C^ in addition to porcine PrP^C^ was able to interfere with the disease caused by the inoculated TSE agent, even though porcine PrP^Sc^ was inoculated (Fig. 3B and Fig. 4B). The highest interference was observed when pig BSE was inoculated into mice coexpressing murine and porcine PrP^C^ (Fig. 3B). In this case, very low infectivity was detected after the second passage, suggesting only propagation of porcine PrP^Sc^. In parallel experiments, coexpression of porcine PrP^C^ was unable to interfere with the disease caused by the inoculation of mouse BSE in heterozygous TgMo/TgPo mice (Fig. 3A). In other words, the homologous replication of murine PrP^Sc^ was not affected by the presence of the inconvertible heterologous porcine PrP^C^, in the same way observed with heterologous bovine PrP^C^ in Fig. 1A. However, expression of porcine PrP^C^ even at lower levels than bovine PrP^C^ interferes with the disease caused by the inoculation of cattle BSE in heterozygous TgBo/TgPo mice (Fig. 4A). Moreover, when cattle BSE is inoculated into mice coexpressing murine and porcine PrP^C^ (that is a PrP^Sc^ heterologous to both PrP^C^ amino acid sequences expressed in the host), only murine PrP^Sc^ is generated after a long survival time in only 40% of the mice (Fig. 6A). We can speculate that porcine PrP^C^ might inhibit (in a competitive manner) the interaction of the murine PrP^C^ with cellular ligands or host factors required only for the propagation process of the heterologous conversion of murine PrP^C^ to PrP^Sc^ (10) but not for the homologous conversion. In this sense, we cannot exclude the role of host-specific factors implicated in the formation of murine PrP^Sc^, as factors other than PrP can affect the infectious process (31, 32). On the other hand, when bovine and porcine PrP^C^ are coexpressed (Fig. 4), both PrP^C^ sequences are not in their natural hosts (cattle or pig), and mutual interference is observed, as neither bovine nor porcine PrP^Sc^ can overcome the interference in terms of survival time or infectivity while infectivity from both PrP^Sc^ species is generated.

Collectively, our results support the idea that the prion replication interference induced by the coexpression of a heterologous PrP^C^ may be related to the conversion susceptibility of the interfering PrP^C^. Bovine PrP^C^ can be converted efficiently by the different prion strains used (see reference 23 and this work), and hence, in the heterozygous transmissions where bovine PrP would interfere, low interference scores were observed (IS < 1.82). The effective convertibility of bovine PrP^C^ by the different prion strains used would explain its poor, if any, interfering effect, allowing the propagation of either mouse or porcine-PrP^Sc^. Conversely, the limited convertibility of porcine PrP^C^ (see references 39 and 40 and this work) would explain the substantial interference effect caused by the coexpression of porcine PrP^C^ with either bovine or murine PrP^C^ in most inocula used, showing interference scores over 2.08. The only exception was the homologous propagation of mouse BSE in TgMo/TgPo mice (Fig. 3A), which was probably due to the effect of host-specific factors involved in the interference process, as mentioned before. Alternatively, specific structural elements in the mouse PrP^Sc^ absent in both cattle and pig BSE PrP^Sc^ could explain the ability of the homologous propagation of mouse BSE in heterozygous mice (TgMo/TgPo and TgMo/TgBo), avoiding the interference effect caused by the coexpression of bovine or porcine PrP^C^. Curiously, while PrP^Sc^ was not detected in hemizygous mice expressing only porcine PrP^C^ inoculated with cattle BSE (Fig. 4A), mice coexpressing porcine and bovine PrP^C^ were able to efficiently propagate porcine PrP^Sc^, as confirmed via its second passage. Porcine PrP^Sc^ replication is likely the result of its interaction with the replicated bovine PrP^Sc^, which would provide a steady source of bovine PrP^Sc^ to interact with porcine PrP, but not with the inoculated bovine PrP^Sc^.

In the cases of the sheep isolates used in Fig. 6B and C, the amino acid sequence of the inoculated PrP^Sc^ (sheep) was different from those of both PrP^C^ expressed in the host (mouse and cattle). Several factors may participate in the transmission of the inoculated TSE agent when three different PrP amino acid sequences are implicated: (i) the transmission barrier of each PrP^C^ to the inoculated PrP^Sc^, (ii) the differential ability of each PrP^C^ to replicate the inoculated prion strain, and (iii) the interference effect of each PrP^C^ on the replication of the other. In this multifaceted scenario, it is difficult to predict the outcome when there is no homology between the inoculum and any of the coexpressed PrP^C^ sequences. In general, the PrP^Sc^ prone to replicate is impaired by the presence of the PrP sequence putatively averse to replicate, as observed when cattle BSE was inoculated into mice coexpressing both porcine and murine PrP^C^ (Fig. 6A), or with sheep BSE or sheep Sc21 inoculated in the different PrP combinations (Fig. 6B to D).

Taken together, all of our results suggest that the coexpression of a PrP^C^ from a second species would interfere with propagation of the homologous prion. The level of interference is generally related to the transmission proficiency of the infectious agent when this second PrP^C^ is expressed alone. That is, effective interference was observed when the inoculated prion was not (or poorly) transmitted in mice expressing the interfering PrP^C^ alone, thus suggesting a certain correlation between interference ability and conversion incompetence of the interfering PrP^C^. Although most of the results supporting this statement have been obtained with classical BSE, results with other prion agents, such as sheep scrapie and L-type BSE, suggest that this contention can be extended to other prion agents, being probably a general rule applying to the different prion strains. This rationale is consistent with the stone fence model (43), which predicts that for a given TSE agent, a conversion-incompetent PrP^C^ will impair the PrP^Sc^ replication of a conversion-competent PrP^C^, resulting in a lower efficacy of prion propagation. As illustrated here, this lower efficiency is translated to reduced attack rates and/or prolonged survival times due to a dominant-negative effect induced by the conversion-incompetent PrP^C^ on a strain-dependent basis. The protector effect of the Val_129_ human PrP variant in heterozygosis for both classical BSE and L-type BSE infection is an example of this dominant-negative effect (44, 45). Finally, the unequal interference capacity of the murine PrP allele, which is expressed in its natural context (the mouse), allows us to speculate that host-specific factors other than PrP could be involved in the interference process.

## MATERIALS AND METHODS

### Ethics statement.

Animal experiments were carried out in strict accordance with institutional and national guidelines and in accordance with the European Directives 86/609/EEC and 2010/63/EU. Every effort was made to minimize animal suffering. The animal experiments conducted at CISA-INIA (Centro de Investigación en Sanidad Animal) were approved by the Committee on the Ethics of Animal Experiments of the Instituto Nacional de Investigación y Tecnología Agraria y Alimentaria (permit no. CEEA 2009/003 and CEEA 2009/004). Experiments developed at ENVT (Ecole Nationale Vétérinaire de Toulouse) were approved by the local ENVT committee (permit no. 01734.01).

### Transgenic mice.

Three transgenic mouse lines previously reported were used: (i) PoPrP-Tg001, expressing porcine PrP^C^ (38); (ii) BoPrP-Tg110, expressing bovine PrP^C^ (46); and (iii) Tga20, expressing murine PrP^C^ (47). PoPrP-Tg001, BoPrP-Tg110, and Tga20 mice are homozygous for each transgene and were generated in a null background for murine PrP (mu*Prnp*^−/−^). PoPrP-Tg001, BoPrP-Tg110, and Tga20 mice are abbreviated in the text as TgPo, TgBo, and TgMo, respectively. These mouse lines were crossbred to obtain heterozygous animals expressing bovine and porcine PrP^C^, bovine and murine PrP^C^, or murine and porcine PrP^C^ (Table 1). As controls, TgPo, TgBo, and TgMo were crossbred with PrP knockout mice (*Prnp*^−/−^) to produce hemizygous animals (Table 1).

### TSE inocula.

All inocula were prepared as 10% brain homogenates in 5% glucose in distilled water. The brain sources were (i) Ca-BSE_0_ French case no. 139, from brainstem of a cow naturally infected with classical BSE; (ii) cattle BSE, from a pool of brains from terminally ill TgBo mice inoculated with Ca-BSE_0_; (iii) pig BSE, from a pool of brains of terminally ill porcine TgPo mice inoculated with a second passage of the Ca-BSE_0_ inoculum; (iv) mouse BSE, from a pool of brains of terminally ill murine transgenic TgMo mice inoculated with a second passage of the Ca-BSE_0_ inoculum; (v) cattle BSE-L, from brainstem of a cow from France naturally infected with L-type atypical BSE; (vi) sheep BSE, from a pool of brains from seven ARQ/ARQ sheep inoculated with Ca-BSE_0_; (vii) sheep Sc21, an isolate obtained from the brain of a French ARQ/ARQ (136, 154, and 171 codons) sheep naturally infected with scrapie; and (viii) as a negative control, a pool of brains of uninoculated C57BL/6 mice.

### Transmission studies.

Groups of 6 to 10 mice (6 to 7 weeks old, weighing approximately 20 g) were anesthetized with isoflurane and inoculated with 2 mg of brain homogenate in the right parietal lobe by using a disposable 25-gauge hypodermic needle. Eight-millimeter transponders were used for individual identification of mice. Mice were examined twice weekly for neurological signs of prion disease and were euthanized by cervical dislocation when the progression of the disease was evident or at the end of the study at 650 days postinoculation (dpi). The animals were humanely euthanized once a definitive diagnosis had been made or earlier if showing signs of distress or loss of up to 20% body weight. A mouse was scored positive for prion disease when it showed 2 or 3 out of 10 described signs of neurological dysfunction (35, 48). Once euthanized, a necropsy was performed, and the brain was harvested and stored at −20°C. Survival time was calculated as the mean ± standard deviation (SD). A Student’s unpaired, two-tailed *t* test was used for comparison between group data (*P* < 0.05). To analyze and compare the levels of interference of prion propagation among the different intracranially inoculated transgenic mice used in the work we introduced a new parameter called the interference score (IS) of the interfering PrP, which takes into consideration both attack rate and survival time. IS was calculated according to the formula

$$\text{IS }=\frac{\text{mean survival time in heterozygous transmission }}{\text{mean survival time in hemizygous transmission }}\times \frac{\text{inoculated animals}}{{\text{diseased PrP}}^{\text{res}}-\text{ positive animals in heterozygous transmission}}$$

If 0 animals were scored PrP^res^ positive in the heterozygous transmission, the IS was calculated considering that the value is higher than when 1 animal would be infected. IS was not calculated if 0 animals were scored positive in the hemizygous transmission. An IS of around 1 indicates no or little interference in the propagated prion, while values over 1 indicate proportionally higher interference in prion propagation.

### PrP^res^ Western blotting.

A mass of around 175 ± 20 mg of frozen brain tissue was homogenized in 5% glucose in distilled water in grinding tubes (Bio-Rad) and adjusted to 10% (wt/vol) using a TeSeE Precess 48 homogenizer (Bio-Rad) following the manufacturer’s instructions. The presence of PrP^res^ (protease-resistant PrP) was determined by Western blotting (23), following the procedure described below and using the reagents of the enzyme-linked immunosorbent assay (ELISA) commercial test (TeSeE; Bio-Rad). Ten to 100 μl of a 10% (wt/vol) brain homogenate were diluted in 190 to 100 μl of a 10% (wt/vol) homogenate from sheep brain scored negative for PrP^res^, to obtain a 200-μl final volume. Homogenates were incubated for 15 min at 37°C with 200 μl of a 2% proteinase K solution (in buffer A). PrP^res^ was recovered as a pellet after addition of 200 μl of buffer B and centrifugation at 15,000 × *g* for 7 min at 20°C. Supernatants were discarded, and pellets were dried inverted over absorbent paper for 5 min. Pellets were solubilized in Laemmli buffer, and samples were incubated for 5 min at room temperature, solubilized, and heated at 100°C for 5 min. Samples were centrifuged at 20,000 × *g* for 15 min at 20°C and supernatants were recovered and loaded on a 12% Bis-Tris gel (Criterion XT [Bio-Rad] or NuPage [Invitrogen]). Proteins were electrophoretically transferred onto polyvinylidene difluoride (PVDF) or nitrocellulose membranes (Millipore). Membranes were blocked overnight with 2% bovine serum albumin (BSA) blocking buffer. Animals positive for PrP^res^ in their brains were recorded as positive for the disease.

For immunoblotting, the monoclonal antibodies (MAbs) Sha31, SAF83, and 12F10 (49) were used at a concentration of 1 μg/ml. Sha31 recognizes the _156_YEDRYYRE_163_ epitope of the bovine PrP sequence. SAF83 recognizes the epitope between residues 126 and 164 of murine PrP but does not recognize bovine or porcine PrP, and 12F10 recognizes the epitope _155_DYEDRYYRE_163_ of bovine PrP (and porcine PrP) but does not recognize murine PrP. Immunocomplexes were detected by incubating the membranes for 1 h with horseradish peroxidase-conjugated anti-mouse IgG (Amersham Pharmacia Biotech). Immunoblots were developed with enhanced chemiluminescence using Pierce ECL enhanced chemiluminescence Western blotting substrate (Thermo Scientific, Rockford, IL, USA). Images were captured using the ChemiDoc XRS+ system. Densitometric analysis was performed using Image Lab 6.0.1 software.

## References

[1] Griffith JS. 1967. Self-replication and scrapie. Nature 215:1043–1044. doi:10.1038/2151043a0. 4964084

[2] Prusiner SB. 1982. Novel proteinaceous infectious particles cause scrapie. Science 216:136–144. doi:10.1126/science.6801762. 6801762

[3] Pan KM, Baldwin M, Nguyen J, Gasset M, Serban A, Groth D, Mehlhorn I, Huang Z, Fletterick RJ, Cohen FE. 1993. Conversion of alpha-helices into beta-sheets features in the formation of the scrapie prion proteins. Proc Natl Acad Sci U S A 90:10962–10966. doi:10.1073/pnas.90.23.10962. 7902575 PMC47901

[4] Meyer RK, McKinley MP, Bowman KA, Braunfeld MB, Barry RA, Prusiner SB. 1986. Separation and properties of cellular and scrapie prion proteins. Proc Natl Acad Sci U S A 83:2310–2314. doi:10.1073/pnas.83.8.2310. 3085093 PMC323286

[5] Prusiner SB. 1998. Prions. Proc Natl Acad Sci U S A 95:13363–13383. doi:10.1073/pnas.95.23.13363. 9811807 PMC33918

[6] Cronier S, Gros N, Tattum MH, Jackson GS, Clarke AR, Collinge J, Wadsworth JD. 2008. Detection and characterization of proteinase K-sensitive disease-related prion protein with thermolysin. Biochem J 416:297–305. doi:10.1042/BJ20081235. 18684106 PMC2584334

[7] Safar J, Wille H, Itri V, Groth D, Serban H, Torchia M, Cohen FE, Prusiner SB. 1998. Eight prion strains have PrP(Sc) molecules with different conformations. Nat Med 4:1157–1165. doi:10.1038/2654. 9771749

[8] Collinge J, Clarke AR. 2007. A general model of prion strains and their pathogenicity. Science 318:930–936. doi:10.1126/science.1138718. 17991853

[9] Bessen RA, Marsh RF. 1992. Biochemical and physical properties of the prion protein from two strains of the transmissible mink encephalopathy agent. J Virol 66:2096–2101. doi:10.1128/JVI.66.4.2096-2101.1992. 1347795 PMC289000

[10] Priola SA, Caughey B, Race RE, Chesebro B. 1994. Heterologous PrP molecules interfere with accumulation of protease-resistant PrP in scrapie-infected murine neuroblastoma cells. J Virol 68:4873–4878. doi:10.1128/JVI.68.8.4873-4878.1994. 7913509 PMC236427

[11] Holscher C, Delius H, Burkle A. 1998. Overexpression of nonconvertible PrPc delta114–121 in scrapie-infected mouse neuroblastoma cells leads to trans-dominant inhibition of wild-type PrP(Sc) accumulation. J Virol 72:1153–1159. doi:10.1128/JVI.72.2.1153-1159.1998. 9445012 PMC124590

[12] Haldiman T, Kim C, Cohen Y, Chen W, Blevins J, Qing L, Cohen ML, Langeveld J, Telling GC, Kong Q, Safar JG. 2013. Co-existence of distinct prion types enables conformational evolution of human PrPSc by competitive selection. J Biol Chem 288:29846–29861. doi:10.1074/jbc.M113.500108. 23974118 PMC3795283

[13] Wells GA, Scott AC, Johnson CT, Gunning RF, Hancock RD, Jeffrey M, Dawson M, Bradley R. 1987. A novel progressive spongiform encephalopathy in cattle. Vet Rec 121:419–420. doi:10.1136/vr.121.18.419. 3424605

[14] Bruce ME, Will RG, Ironside JW, McConnell I, Drummond D, Suttie A, McCardle L, Chree A, Hope J, Birkett C, Cousens S, Fraser H, Bostock CJ. 1997. Transmissions to mice indicate that ‘new variant’ CJD is caused by the BSE agent. Nature 389:498–501. doi:10.1038/39057. 9333239

[15] Collinge J, Rossor M. 1996. A new variant of prion disease. Lancet 347:916–917. doi:10.1016/s0140-6736(96)91407-5. 8598749

[16] Hill AF, Desbruslais M, Joiner S, Sidle KC, Gowland I, Collinge J, Doey LJ, Lantos P. 1997. The same prion strain causes vCJD and BSE. Nature 389:448–450, 526. doi:10.1038/38925. 9333232

[17] Eiden M, Hoffmann C, Balkema-Buschmann A, Muller M, Baumgartner K, Groschup MH. 2010. Biochemical and immunohistochemical characterization of feline spongiform encephalopathy in a German captive cheetah. J Gen Virol 91:2874–2883. doi:10.1099/vir.0.022103-0. 20660146

[18] Kirkwood JK, Cunningham AA. 1994. Epidemiological observations on spongiform encephalopathies in captive wild animals in the British Isles. Vet Rec 135:296–303. doi:10.1136/vr.135.13.296. 7817514

[19] Seuberlich T, Botteron C, Wenker C, Café-Marçal VA, Oevermann A, Haase B, Leeb T, Heim D, Zurbriggen A. 2006. Spongiform encephalopathy in a miniature zebu. Emerg Infect Dis 12:1950–1953. doi:10.3201/eid1212.060750. 17326950 PMC3291368

[20] Eloit M, Adjou K, Coulpier M, Fontaine JJ, Hamel R, Lilin T, Messiaen S, Andreoletti O, Baron T, Bencsik A, Biacabe AG, Beringue V, Laude H, Le Dur A, Vilotte JL, Comoy E, Deslys JP, Grassi J, Simon S, Lantier F, Sarradin P. 2005. BSE agent signatures in a goat. Vet Rec 156:523–524. doi:10.1136/vr.156.16.523-b. 15833975

[21] Jeffrey M, Martin S, Gonzalez L, Foster J, Langeveld JP, van Zijderveld FG, Grassi J, Hunter N. 2006. Immunohistochemical features of PrP(d) accumulation in natural and experimental goat transmissible spongiform encephalopathies. J Comp Pathol 134:171–181. doi:10.1016/j.jcpa.2005.10.003. 16542672

[22] Bruce ME. 2003. TSE strain variation. Br Med Bull 66:99–108. doi:10.1093/bmb/66.1.99. 14522852

[23] Torres JM, Espinosa JC, Aguilar-Calvo P, Herva ME, Relano-Gines A, Villa-Diaz A, Morales M, Parra B, Alamillo E, Brun A, Castilla J, Molina S, Hawkins SA, Andreoletti O. 2014. Elements modulating the prion species barrier and its passage consequences. PLoS One 9:e89722. doi:10.1371/journal.pone.0089722. 24608126 PMC3946430

[24] Casalone C, Zanusso G, Acutis P, Ferrari S, Capucci L, Tagliavini F, Monaco S, Caramelli M. 2004. Identification of a second bovine amyloidotic spongiform encephalopathy: molecular similarities with sporadic Creutzfeldt-Jakob disease. Proc Natl Acad Sci U S A 101:3065–3070. doi:10.1073/pnas.0305777101. 14970340 PMC365745

[25] Biacabe AG, Laplanche JL, Ryder S, Baron T. 2004. Distinct molecular phenotypes in bovine prion diseases. EMBO Rep 5:110–115. doi:10.1038/sj.embor.7400054. 14710195 PMC1298965

[26] Benestad SL, Sarradin P, Thu B, Schonheit J, Tranulis MA, Bratberg B. 2003. Cases of scrapie with unusual features in Norway and designation of a new type, Nor98. Vet Rec 153:202–208. doi:10.1136/vr.153.7.202. 12956297

[27] Bruce ME, McConnell I, Fraser H, Dickinson AG. 1991. The disease characteristics of different strains of scrapie in Sinc congenic mouse lines: implications for the nature of the agent and host control of pathogenesis. J Gen Virol 72:595–603. doi:10.1099/0022-1317-72-3-595. 1672371

[28] Langeveld JPM, Pirisinu L, Jacobs JG, Mazza M, Lantier I, Simon S, Andreoletti O, Acin C, Esposito E, Fast C, Groschup M, Goldmann W, Spiropoulos J, Sklaviadis T, Lantier F, Ekateriniadou L, Papasavva-Stylianou P, van Keulen LJM, Acutis PL, Agrimi U, Bossers A, Nonno R. 2019. Four types of scrapie in goats differentiated from each other and bovine spongiform encephalopathy by biochemical methods. Vet Res 50:97. doi:10.1186/s13567-019-0718-z. 31767033 PMC6878695

[29] Nonno R, Marin-Moreno A, Carlos Espinosa J, Fast C, Van Keulen L, Spiropoulos J, Lantier I, Andreoletti O, Pirisinu L, Di Bari MA, Aguilar-Calvo P, Sklaviadis T, Papasavva-Stylianou P, Acutis PL, Acin C, Bossers A, Jacobs JG, Vaccari G, D’Agostino C, Chiappini B, Lantier F, Groschup MH, Agrimi U, Maria Torres J, Langeveld JPM. 2020. Characterization of goat prions demonstrates geographical variation of scrapie strains in Europe and reveals the composite nature of prion strains. Sci Rep 10:19. doi:10.1038/s41598-019-57005-6. 31913327 PMC6949283

[30] Beringue V, Vilotte JL, Laude H. 2008. Prion agent diversity and species barrier. Vet Res 39:47. doi:10.1051/vetres:2008024. 18519020

[31] Crowell J, Hughson A, Caughey B, Bessen RA. 2015. Host determinants of prion strain diversity independent of prion protein genotype. J Virol 89:10427–10441. doi:10.1128/JVI.01586-15. 26246570 PMC4580196

[32] Espinosa JC, Nonno R, Di Bari M, Aguilar-Calvo P, Pirisinu L, Fernandez-Borges N, Vanni I, Vaccari G, Marin-Moreno A, Frassanito P, Lorenzo P, Agrimi U, Torres JM. 2016. PrPC governs susceptibility to prion strains in bank vole, while other host factors modulate strain features. J Virol 90:10660–10669. doi:10.1128/JVI.01592-16. 27654300 PMC5110189

[33] Groschup MH, Buschmann A. 2008. Rodent models for prion diseases. Vet Res 39:32. doi:10.1051/vetres:2008008. 18284909

[34] Watts JC, Prusiner SB. 2014. Mouse models for studying the formation and propagation of prions. J Biol Chem 289:19841–19849. doi:10.1074/jbc.R114.550707. 24860095 PMC4106304

[35] Scott M, Foster D, Mirenda C, Serban D, Coufal F, Wälchli M, Torchia M, Groth D, Carlson G, DeArmond SJ, Westaway D, Prusiner SB. 1989. Transgenic mice expressing hamster prion protein produce species-specific scrapie infectivity and amyloid plaques. Cell 59:847–857. doi:10.1016/0092-8674(89)90608-9. 2574076

[36] Prusiner SB, Scott M, Foster D, Pan KM, Groth D, Mirenda C, Torchia M, Yang SL, Serban D, Carlson GA. 1990. Transgenetic studies implicate interactions between homologous PrP isoforms in scrapie prion replication. Cell 63:673–686. doi:10.1016/0092-8674(90)90134-z. 1977523

[37] Telling GC, Scott M, Mastrianni J, Gabizon R, Torchia M, Cohen FE, DeArmond SJ, Prusiner SB. 1995. Prion propagation in mice expressing human and chimeric PrP transgenes implicates the interaction of cellular PrP with another protein. Cell 83:79–90. doi:10.1016/0092-8674(95)90236-8. 7553876

[38] Castilla J, Gutierrez-Adan A, Brun A, Doyle D, Pintado B, Ramirez MA, Salguero FJ, Parra B, Diaz San Segundo F, Sanchez-Vizcaino JM, Rogers M, Torres JM. 2004. Subclinical bovine spongiform encephalopathy infection in transgenic mice expressing porcine prion protein. J Neurosci 24:5063–5069. doi:10.1523/JNEUROSCI.5400-03.2004. 15163699 PMC6729370

[39] Espinosa JC, Herva ME, Andreoletti O, Padilla D, Lacroux C, Cassard H, Lantier I, Castilla J, Torres JM. 2009. Transgenic mice expressing porcine prion protein resistant to classical scrapie but susceptible to sheep bovine spongiform encephalopathy and atypical scrapie. Emerg Infect Dis 15:1214–1221. doi:10.3201/eid1508.081218. 19751582 PMC2815954

[40] Espinosa JC, Marín-Moreno A, Aguilar-Calvo P, Benestad SL, Andreoletti O, Torres JM. 2020. Porcine prion protein as a paradigm of limited susceptibility to prion strain propagation. J Infect Dis 221:2085–2085. doi:10.1093/infdis/jiz646. 32119741 PMC7289552

[41] Caughey B. 2003. Prion protein conversions: insight into mechanisms, TSE transmission barriers and strains. Br Med Bull 66:109–120. doi:10.1093/bmb/66.1.109. 14522853

[42] Horiuchi M, Priola SA, Chabry J, Caughey B. 2000. Interactions between heterologous forms of prion protein: binding, inhibition of conversion, and species barriers. Proc Natl Acad Sci U S A 97:5836–5841. doi:10.1073/pnas.110523897. 10811921 PMC18520

[43] Kobayashi A, Hizume M, Teruya K, Mohri S, Kitamoto T. 2009. Heterozygous inhibition in prion infection: the stone fence model. Prion 3:27–30. doi:10.4161/pri.3.1.8514. 19372732 PMC2676740

[44] Fernandez-Borges N, Espinosa JC, Marin-Moreno A, Aguilar-Calvo P, Asante EA, Kitamoto T, Mohri S, Andreoletti O, Torres JM. 2017. Protective effect of Val129-PrP against bovine spongiform encephalopathy but not variant Creutzfeldt-Jakob disease. Emerg Infect Dis 23:1522–1530. doi:10.3201/eid2309.161948. 28820136 PMC5572891

[45] Marin-Moreno A, Hour A, Espinosa JC, Douet JY, Aguilar-Calvo P, Lacroux C, Piquer J, Lugan S, Lorenzo P, Tillier C, Aron N, Cassard H, Andreoletti O, Torres JM. 2020. Radical change in zoonotic abilities of atypical BSE prion strains as evidenced by crossing of sheep species barrier in transgenic mice. Emerg Infect Dis J 26:1130–1139. doi:10.3201/eid2606.181790. PMC7258450 32441630

[46] Castilla J, Gutierrez Adan A, Brun A, Pintado B, Ramirez MA, Parra B, Doyle D, Rogers M, Salguero FJ, Sanchez C, Sanchez-Vizcaino JM, Torres JM. 2003. Early detection of PrP(res) in BSE-infected bovine PrP transgenic mice. Arch Virol 148:677–691. doi:10.1007/s00705-002-0958-4. 12664293

[47] Fischer M, Rulicke T, Raeber A, Sailer A, Moser M, Oesch B, Brandner S, Aguzzi A, Weissmann C. 1996. Prion protein (PrP) with amino-proximal deletions restoring susceptibility of PrP knockout mice to scrapie. EMBO J 15:1255–1264. doi:10.1002/j.1460-2075.1996.tb00467.x. 8635458 PMC450028

[48] Scott M, Groth D, Foster D, Torchia M, Yang SL, DeArmond SJ, Prusiner SB. 1993. Propagation of prions with artificial properties in transgenic mice expressing chimeric PrP genes. Cell 73:979–988. doi:10.1016/0092-8674(93)90275-u. 8098995

[49] Feraudet C, Morel N, Simon S, Volland H, Frobert Y, Creminon C, Vilette D, Lehmann S, Grassi J. 2005. Screening of 145 anti-PrP monoclonal antibodies for their capacity to inhibit PrPSc replication in infected cells. J Biol Chem 280:11247–11258. doi:10.1074/jbc.M407006200. 15618225

[50] Manson JC, Clarke AR, Hooper ML, Aitchison L, McConnell I, Hope J. 1994. 129/Ola mice carrying a null mutation in PrP that abolishes mRNA production are developmentally normal. Mol Neurobiol 8:121–127. doi:10.1007/BF02780662. 7999308

