# Supplementary figures and images for: Allelic Interference in Prion Replication Is Modulated by the Convertibility of the Interfering PrPC and Other Host-Specific Factors
Source: mBio. 2021 Mar 16;12(2):e03508-20. doi: 10.1128/mBio.03508-20 (PMC8092304; doi:10.1128/mBio.03508-20)

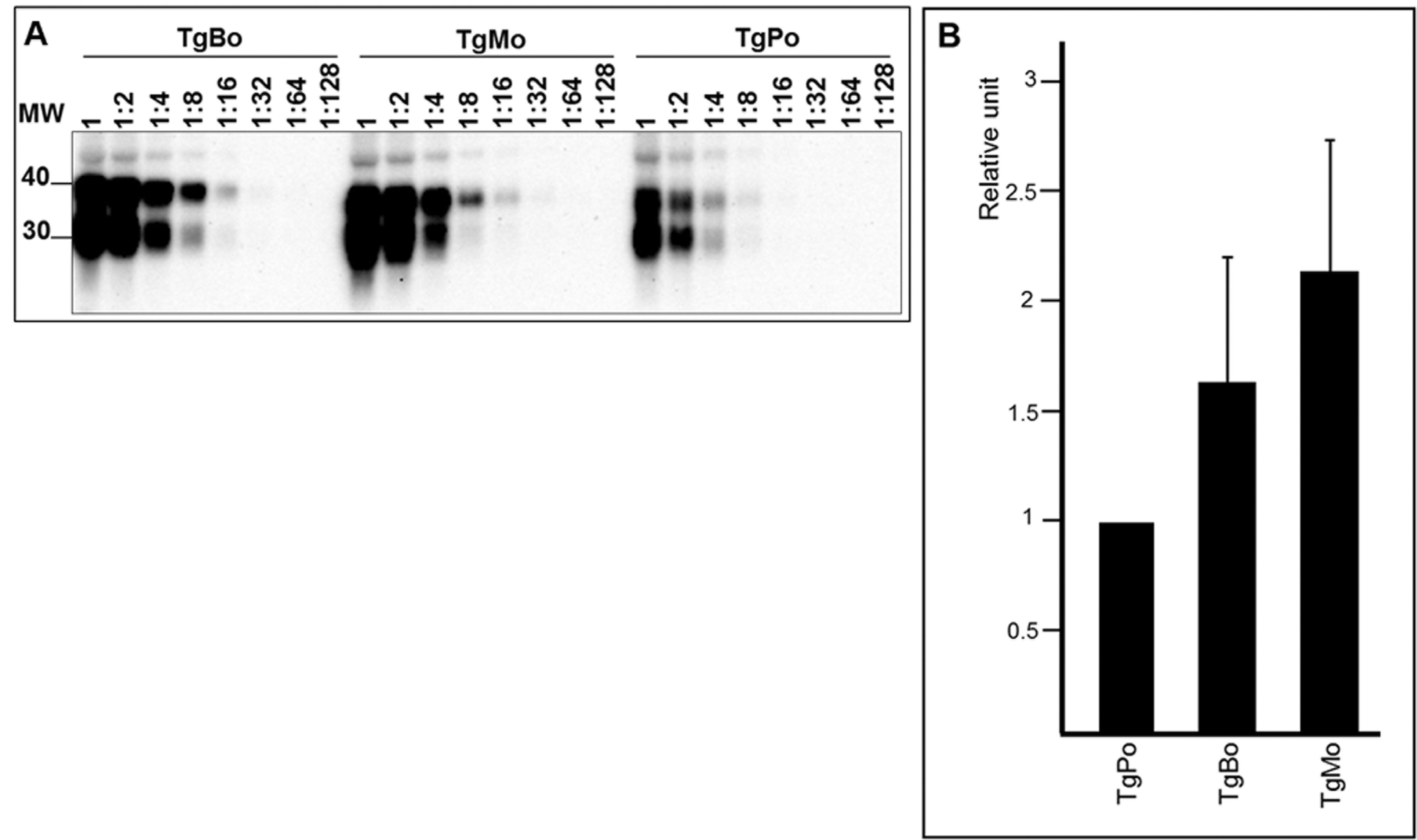

Supplement: FIG S1 [file mBio.03508-20-sf001.tif]
